# Atypical Porcine Pestivirus: A Possible Cause of Congenital Tremor Type A-II in Newborn Piglets

**DOI:** 10.3390/v8100271

**Published:** 2016-10-04

**Authors:** Ad de Groof, Martin Deijs, Lars Guelen, Lotte van Grinsven, Laura van Os-Galdos, Wannes Vogels, Carmen Derks, Toine Cruijsen, Victor Geurts, Mieke Vrijenhoek, Janneke Suijskens, Peter van Doorn, Leo van Leengoed, Carla Schrier, Lia van der Hoek

**Affiliations:** 1 MSD Animal Health/Intervet International bv., Department Discovery & Technology, Wim de Körverstraat 35, P.O. Box 31, 5830AA Boxmeer, The Netherlands; LGuelen@aduro.com (L.G.); lotte.van.grinsven@merck.com (L.v.G.); wannes.vogels@merck.com (W.V.); carmen.derks@merck.com (C.D.); mieke.vrijenhoek@merck.com (M.V.); jannekesuijskens@hotmail.com (J.S.); petervandoorn@gmail.com (P.v.D.); carla.schrier@vaxxinova.com (C.S.); 2 Laboratory of Experimental Virology, Department of Medical Microbiology, Center for Infection and Immunity Amsterdam (CINIMA), Academic Medical Center, University of Amsterdam, 1105AZ, Amsterdam, The Netherlands; m.deijs@amc.uva.nl (M.D.); laura_v1310@hotmail.com (L.v.O.-G.); 3 MSD Animal Health/Intervet Nederland bv., Wim de Körverstraat 35, P.O. Box 31, 5830AA Boxmeer, The Netherlands; toine.cruijsen@merck.com (T.C.); victor.geurts@merck.com (V.G.); 4 Department of Farm Animal Health, Faculty of Veterinary Medicine, University of Utrecht, 3584CL, Utrecht, The Netherlands; l.vanleengoed@uu.nl

**Keywords:** pestivirus, congenital tremor, swine, persistent infection, APPV

## Abstract

Congenital tremor type A-II in piglets has been regarded as a transmissible disease since the 1970s, possibly caused by a very recently-described virus: atypical porcine pestivirus (APPV). Here, we describe several strains of APPV in piglets with clinical signs of congenital tremor (10 of 10 farms tested). Piglets on a farm with no history of congenital tremor were PCR-negative for the virus. To demonstrate a causal relationship between APPV and disease, three gilts were inoculated via intramuscular injection at day 32 of pregnancy. In two of the three litters, vertical transmission of the virus occurred. Clinical signs of congenital tremor were observed in APPV-infected newborns, yet also two asymptomatic carriers were among the offspring. Piglets of one litter were PCR-negative for the virus, and these piglets were all without congenital tremors. Long-term follow up of farm piglets born with congenital tremors showed that the initially high viremia in serum declines at five months of age, but shedding of the virus in feces continues, which explains why the virus remains present at affected farms and causes new outbreaks. We conclude that trans-placental transmission of APPV and subsequent infection of the fetuses is a very likely cause of congenital tremor type A-II in piglets.

## 1. Introduction

Congenital tremor is a well-known phenomenon in newborn piglets [1], which was first described in 1922, when ‘dancing pigs’ were described by Kinsley [2]. Over the course of nearly a century, several articles have been published that describe the same clinical signs under varying names, including shaking pig disease, tremor in pigs, and myoclonia congenita [3,4]. Congenital tremor is characterized by tremors of the head and limbs that vary in severity, but are reducing or even absent during sleep. These tremors can be aggravated by stress, for example excitement and cold. They last for several weeks to months, but most piglets are clinically normal at weaning age, although in some severely-affected animals, tremors persist into adult life [5]. Although the shaking itself does not directly cause death, the tremors can prevent the piglets from finding a teat to suckle, which can subsequently cause severe growth retardation or death by starvation. Congenital tremors are often complicated with splayed hind legs [6].

Historically, congenital tremor has been classified as type A or type B [1,7]. Type A comprises the cases with visible histological lesions in the brain and spinal cord, whereas the cases of type B display no apparent lesions. Type A is further divided into five subgroups, based on the different causes of congenital tremor. Type A-I cases of congenital tremor are caused by classical swine fever virus (CSFV) and are characterized by cerebellar hypoplasia [8,9,10]. The cause of type A-III congenital tremor is a genetic (sex-linked) defect existing only in the Landrace breed, which is characterized by the absence of tight myelin sheaths and less oligodendrocytes [11], while a recessive genetic (autosomal-linked) defect in the Saddleback breed is the cause of type A-IV, which is characterized by cerebro-spinal hypomyelinogenesis [6,12,13]. Type A-V cases are caused by trichlorfon toxicosis, an intoxication that is often linked to organophosphorus-treated food [12]. These are characterized by cerebellar hypoplasia [12,14]. Type A-II cases have been the most puzzling cases [1,5,7].

The assumption is that type A-II congenital tremor is caused by an infectious agent, as the clinical signs can be reproduced experimentally. Some 40 years ago, Patterson et al. [5] managed to induce type A-II congenital tremor in piglets through experimental infection of pregnant sows with a homogenate of spinal cord, brains and spleens of clinically-affected piglets. Furthermore, most of the type A-II shaking piglets are born from gilts (female pigs in their first pregnancy) that have recently been introduced into a new environment [6]. Notably, after a first litter with shaking piglets, subsequent litters of the same sow hardly ever show signs of congenital tremor [6]. This is an indication that immunity develops, protecting against the suspected infectious agent that causes congenital tremor.

Very recently it has been shown that a novel porcine pestivirus is likely involved in type A-II congenital tremor [15]. This novel pestivirus has been first described by Hause et al. [16]: atypical porcine pestivirus (APPV). Arruda et al. [15] inoculated the virus in pregnant sows and the fetal amniotic vesicles of these sows. The sows did not get infected by APPV, yet the birth of piglets with congenital tremor after APPV inoculation in the amniotic fluid provided a strong indication that congenital tremor type A-II is caused by APPV [15].

Since 2012, we monitored outbreaks of congenital tremor type A-II in the Netherlands. Clinical samples from the outbreaks all contained APPV. Characterization of the strains showed large genetic variation (>10% nt difference) and at least three clusters. The question of whether a natural route of infection, being vertical transmission, causes congenital tremor type A-II in the offspring was addressed by infection of gilts during gestation and monitoring of the offspring. Finally, we examined whether infected and surviving farm piglets become persistent shedders of the virus, which may explain the high recurrence rate of congenital tremor type A-II at affected farms.

## 2. Materials and Methods

### 2.1. Ethics Statement

All animal experimental procedures were carried out in the Netherlands at MSD Animal Health (MSD-AH) and performed in strict accordance with the specific regulations that govern animal research in the Netherlands, following the Guidelines set forth by the Dutch government in the Experiments on Animals Act (WOD, 2014). MSD-AH is audited annually and licensed by the Netherlands Food and Consumer Product Safety Authority (NVWA) to perform animal research (License No. MSD-AH Boxmeer is 22100). The MSD-AH animal ethics committee reviewed and approved the animal care and use protocol (ADT 14.023).

### 2.2. Sample Collection

Fecal and serum samples were obtained from farms in the Netherlands (Farms 1–10) and one farm in Spain (Farm 11). Blood was collected using Vacuolette 5/8 mL Sep Clot Activator (Greiner-Bio One, Kremsmünster, Austria) and serum obtained by centrifugation 10 min at 3200× *g* at 4 °C. Rectal swabs were collected using a dry cotton swab and put in a sterile tube containing 2 mL phosphate-buffered saline solution (PBS). Subsequently, the cotton swabs were stirred strongly and discarded. Both serum and fecal samples were stored at −70 °C until analysis. Peripheral blood lymphocytes (PBLs) were collected from citrate-phosphate-dextrose-adenine (CPDA) containing blood samples after Leucosep (Greiner-Bio One) density gradient centrifugation.

Postmortem examination was performed on piglets from outbreaks of congenital tremor type A-II and on control piglets without clinical signs. Organs were sampled and frozen at −70 °C until analysis. Homogenates (10%) were made in PBS on ice. Homogenization was performed in Gentle Macs M tubes with the Gentle Macs Dissociator (Miltenyi Biotec, Bergisch Gladbach, Germany). This homogenized material was then freeze-thawed and centrifuged twice, first at 3200× *g* for 30 min at 4 °C and subsequently at 10,000× *g* for 10 min at 4 °C. Subsequently, a DNase treatment was performed with Turbo DNase (Thermo Fisher Scientific, Waltham, MA, USA).

To determine the association with disease, the following serum samples were collected: 99 samples from newborns with disease (10 different farms), all in their first week of life; 59 serum samples from piglets without clinical signs, but from affected farms (also in their first week of life); seven healthy piglets born in a farm without any history of congenital tremor (sampled in their first week of life). Persistent shedding was investigated in clinical material of 34 additional piglets that were APPV PCR-positive in their first week of life at Farm 6. Serum was collected until 4.5 months of age (29 of the 34) or until 8.5 months of age (5 of the 34). Of the five that were followed until age 8.5 months, also sequential fecal samples were collected. To investigate organ distribution, the following organs were collected from seven APPV PCR-positive, precolostral newborn affected piglets of Farm 1: heart, small intestine, large intestine, brain, thoracic spinal cord, lumbar spinal cord, liver, inguinal lymph node, lung, gallbladder, urinary bladder, kidney, tonsil, spleen and PBLs.

### 2.3. VIDISCA-Next Generation Sequencing

Eleven serum samples of piglets with disease (March/April 2012) and 12 samples without disease (July 2012) were tested by next generation sequencing. All were collected from Farm 1. The VIDISCA-454 was performed as described by de Vries et al. [17]. In short, serum was centrifuged for 10 min at 10,000× *g*, and the supernatant was treated with Turbo DNase (Ambion). Subsequently, nucleic acids were extracted by the Boom extraction method [18]. A reverse transcription reaction with Superscript II (Thermo Fisher Scientific) was performed using non-ribosomal random hexamers [19]. A second strand DNA synthesis was performed with 5 U of Klenow fragment (3′ to 5′ exo minus, New England Biolabs, Ipswich, MA, USA). Double-stranded DNA was purified by phenol/chloroform/isoamyl alcohol extraction and ethanol precipitation and digested with either MseI, Csp6I or CviAII restriction enzyme (New England Biolabs). Adaptors with different multiplex identifier sequences (MIDs) were ligated to the digested fragments of the different samples. Before PCR amplification, the fragments were purified with AMPure XP beads (Agencourt AMPure XP PCR, Beckman Coulter, Brea, CA, USA). Next, a 28-cycle PCR with adaptor-annealing primers was performed. The program of the PCR-reaction was: 5 min at 95 °C and cycles of 1 min at 95 °C, 1 min at 55 °C and 2 min at 72 °C, followed by 10 min at 72 °C and 10 min 4 °C. After purification with AMPure XP beads, the purified DNA was quantified with the Quant-it dsDNA HS Qubit kit (Thermo Fisher Scientific) and diluted to 10^7^ copies/μL. Samples were pooled, and Kapa PCR (Kapa Biosystems, Wilmington, MA, USA) was performed to determine the quantity of DNA in each pool. The Bioanalyser (hsDNA chip, Beckman Coulter) was used to determine the average nucleotide length of the libraries. For Roche-454 sequencing, the pools were diluted until 10^6^ copies/μL, titrated with beads (DNA:beads ratio of 0.5:1, 1:1, 2:1 and 4:1) and used in an emulsion PCR according to the supplier’s protocol (LIB-A SV emPCR kit). Sequencing was done on a 2 region GS FLX Titanium PicoTiterPlate (70 × 75) with the GS FLX Titanium XLR 70 Sequencing kit (Roche, Bradford, CT, USA). Sequence reads were analyzed using the blastn and blastp algorithms (National Center for Biotechnology Information, Bethesda, MD, USA). The library preparation for ion proton next generation sequencing was identical as the library preparation for VIDISCA-454. Following size determination and DNA copy calculations, 50 pM DNA were clonally amplified on beads using the Ion Chef System (Thermo Fisher Scientific). Sequencing was performed on an Ion Proton™ System (Thermo Fisher Scientific).

### 2.4. Detection of Virus in Clinical Samples by PCR

The QIAamp Viral RNA mini Kit (Qiagen, Venlo, The Netherlands) was used on 140 µL or 200 µL clinical sample (fecal sample, serum sample, PBLs) or tissue homogenate in combination with the RNase free DNase kit (Qiagen). In some cases, viral RNA was isolated using the Magnapure 96 instrument (Roche) with external lysis. Both methods were performed as recommended by the suppliers, and harvested RNA was stored at −70 °C until further use or reverse transcribed immediately using the SuperScript III First-Strand Synthesis System (Invitrogen) according to the manufacturer’s protocol with minor modifications: random hexamers and dNTPs were pre-incubated with RNA at 65 °C (5 min) and subsequently chilled on ice. An RNase H (Invitrogen) treatment was performed to degrade the RNA in RNA-DNA hybrids, prior to PCR. PCRs to detect APPV were performed with SuperTaq Plus (Thermo Fisher Scientific) and various APPV primers, depending on the strain (see Table S1 for primer sequences). A universal quantitative reverse transcription PCR (qRT-PCR) was used to quantitatively detect all strains using primers in the most conserved part of the genome (5′ untranslated region (UTR)) APPV-PAN2-F3-B: CGTGCCCAAAGAGAAATCGG and APPV-PAN2-R3-B: CCGGCACTCTATCAAGCAGT and the Superscript III Platinum SYBR Green One-Step qRT-PCR kit (Thermo Fisher Scientific) on a Bio-Rad CFX96. Specificity of SYBR Green qPCRs was validated by melt curve analysis.

### 2.5. Genome Walking

The viral sequences identified after performing VIDISCA-454 were used as a template for primer design to verify the sequences obtained from the deep sequencing and to perform gap-filling PCRs. To determine the sequence of the termini, 5′RACE (Invitrogen) and 3′RACE were performed. The 3′RACE did not yield PCR products when poly-A-tail priming was performed; therefore, the following reactions were performed: RNA of the virus was ligated to an oligonucleotide (JZH-ligate phosphorylated 5′-CACAGTCCATTGTGATGATAGC-3′) using T4 RNA ligase I (New England Biolabs). cDNA was generated with JZH-reverse primer (JZH-primer: 5′-GCTATCATCACAATGGAC-3′) and Superscript II (Invitrogen) followed by PCR using APPV strain Netherlands 1 (NL1) specific primers together with the JZH reverse primer. The RACE-PCR products and gap-filling PCR products were sequenced using BigDye terminator chemistry (BigDye Terminator v1.1 Cycle Sequencing Kit, Applied Biosystems). Sequences were analyzed using Codoncode Aligner Software (Version 4.0.4). The open reading frame (ORF) in the APPV genome was identified via the ORF finder [20].

### 2.6. Submission of Sequences

The sequences of the APPVs genomes are deposited in GenBank under Accession Number KX929062-KX929075.

### 2.7. Phylogeny

Nucleotide and protein sequence alignments were generated using the multiple sequence alignment tool ClustalW [21,22]. Phylogenetic trees were created with MEGA 6.06 software using the neighbor-joining method, with complete deletion in the case of gaps or insertions [23]. A bootstrap analysis of 500 replicates was performed to provide confidences to the clustering.

### 2.8. Infection of Weaner-Aged Piglets

Eight weaner-aged pigs of approximately 4 weeks of age were housed in the MSD-AH test facility. Within the group, direct animal-animal contact was possible. Two pigs served as contact sentinels; six pigs were infected nasally, orally, subcutaneously and intramuscularly with either a pool of spleen, spinal cord and brain (two animals), spleen, tonsils and brain (two animals) or brain and spinal cord (two animals) homogenate. The inoculums (tissue-homogenate-pools) were obtained from 3 APPV NL1-positive farm-piglets with congenital tremor that did not receive colostrum. The tissue was homogenized using a blender, followed by shaking with small glass beads for 5 min. During homogenizing, organ-pulp was kept on ice. The organ-pulp was centrifuged for 1 h at 3200× *g* at 4 °C. Supernatant was first passed over a 0.45-µm filter and subsequently over a 0.22-µm filter (Millipore, Amsterdam, The Netherlands). The filtered homogenate was stored at −70 °C. Four milliliters were inoculated via the oral route, 2 mL subcutaneous route (2 × 1 mL), 2 mL intramuscular route (2 × 1 mL) and 2 mL intranasal route (1 mL in each nostril). Dose varied between 3.9 × 10^5^ and 8.6 × 10^5^ copies/mL in the 10% homogenate.

All inoculated pigs were observed daily for clinical signs of disease, yet there were no clinical signs of congenital tremor within the 15-day observation period. On days 0, 3, 7, 10 and 14, nasal swaps, oropharyngeal swabs, rectal swabs, PBLs and blood-plasma (CPDA-plasma) were collected. The viral load in plasma was monitored via qPCR, and necropsies were performed on day 11 (3 pigs), day 15 (3 pigs) or day 28 (contact sentinels). At necropsy, brain, spinal cord, spleen, tonsils, nasal-, rectal- and oropharyngeal-swabs, PBLs and serum were collected. Based on the qPCR analysis, a collection date where viral load was still rising was selected: day 11 necropsy serum for infection of the pregnant gilts (Figure S1). To check if the serum to be used as inoculum for infection of gilts contains no other virus than APPV, the sample was analyzed via VIDISCA-Ion Torrent. A total of 100,000 sequence reads were screened. Besides APPV NL1 (297 sequence reads), the material contained astrovirus (31 sequence reads).

### 2.9. Inoculation of Pregnant Gilts

Gilts were inoculated on day 32 of gestation with the APPV NL1-positive serum obtained after weaner-aged piglet infection (see above). The APPV load in the inoculum was 6 × 10^5^ copies/mL. An intramuscular injection of 5 mL inoculum (two injections of 2.5 mL each in the left and right neck side) with filtered (0.22-µm) serum was performed. APPV infection was analyzed at day 10 post-inoculation via the qRT-PCR of serum samples. Furthermore, VIDISCA-ion proton analysis was done on the same samples. The VIDISCA-ion proton run found only APPV NL1 infecting the 3 pregnant pigs (65 sequence reads of the 224,000 reads analyzed), indicating that the astrovirus (also present in the inoculum) did not yield a productive infection. To confirm this, a PCR test on cDNA of serum was performed. A nested PCR with primers 5′-GATATGATCTTCCTTGAGATTC-3′ and 5′-CATCCTCATGTTCAAGTCGAAC-3′ (first PCR) and 5′-CACCAGCATTGCAAGGTGTTCC-3′ and 5′-CACCGGTGTAGCCAGTGTTGG-3′ (nested PCR) specifically designed on the astrovirus present in the inoculum was performed. This PCR was negative for all animals and all piglets, so all three gilts at 10 days post-inoculation, the three gilts at giving birth and all piglets from the three litters. A positive PCR-control (inoculum of the gilts) was included, which was positive.

The offspring was scored for congenital tremor: Score 0: no recognizable tremor/muscle contractions; Score 1: mild tremor: mild involuntary subcutaneous muscle fasciculation in the hind legs; Score 2: moderate to severe tremor (moderate to heavy involuntary muscle fasciculation all over the body).

## 3. Results

### 3.1. Outbreaks of Congenital Tremor Type A-II in Pig Farms and Detection of APPV

An outbreak of congenital tremor type A-II was diagnosed on a pig farm (Farm 1) located in the Netherlands in early 2012. Piglets born from gilts were primarily affected, but also piglets born from higher parity sows occasionally showed clinical signs. Diagnosis was based on clinical observations and subsequent exclusion of congenital tremor types A-I, A-III, A-IV and A-V as the possible cause for disease. In the first 20 weeks of the year 2012, a total of 48 litters with congenital tremor were born from gilts, out of 231 litters born from gilts in total. At the peak of the epidemic, eight weeks after the initial outbreak, 85% (11/13) of the litters born from gilts contained one or more piglets with clinical signs of congenital tremor type A-II. The mortality till weaning was 26% in affected litters (the average mortality measured in 48 litters with disease), compared to 11% (average of 183 healthy litters) in controls. In affected litters, 60% of piglet death was attributable to congenital tremor. The total number of piglets born per litter was not affected. Congenital tremor appeared in both sexes, and prevalence within the litter varied between <10% and 100%. Prior to the outbreak in 2012, congenital tremor was observed in a few litters in November 2009 and December 2010.

**Figure 1:**
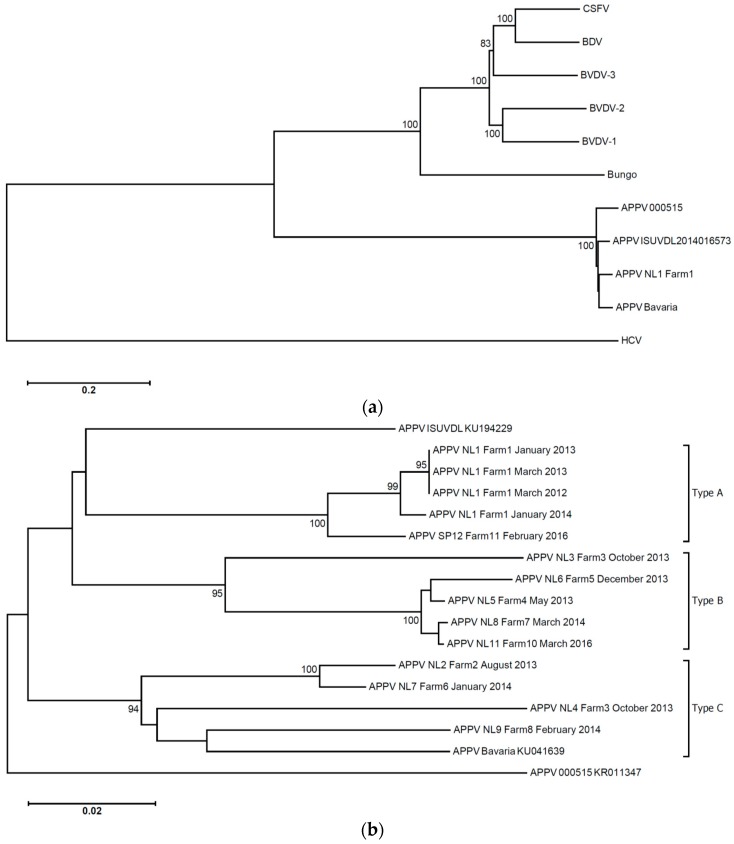
Phylogenetic analysis of the entire coding region (amino acid alignment) of atypical porcine pestivirus (APPV) NL1 Farm1, March 2012, among the known pestiviruses (**a**) and phylogenetic clustering (nucleotide alignment) into various APPV types (**b**). The neighbor-joining method with complete deletion within the MEGA-6.06 package was used; bootstrap values above 80 (for 500 replications) are provided at the root of the clusters. The scale bar is a measure of the proportion of divergence. Hepatitis C virus (NC004102.1) is used as the outgroup in (a). BVDV-3: bovine viral diarrhea virus 3 (NC_012812.1); BDV: border disease virus (NC_003679.1); CSFV: classical swine fever virus (NC_002657.1); BVDV-2: bovine viral diarrhea virus genotype 2 (NC_002032.1); BVDV-1: bovine viral diarrhea virus 1 (NC_001461.1); Bungo: Bungowannah porcine pestivirus (NC_023176.1); APPV 000515: isolate 000515 (KR011347.1); APPV of the Iowa State University Veterinary Diagnostic Laboratory (ISUVDL): ISUVDL2014016573 (KU194229); APPV Bavaria: strain S5/9 (KU041639).

Serum samples were obtained in March 2012 (six piglets with clinical signs of congenital tremor type A-II) and April 2012 (five piglets with clinical signs of congenital tremor type A-II). Sows on the index farm were not sampled. VIDISCA-454, a next generation sequencing technique with unbiased viral RNA amplification, was performed on the serum. The sequence reads showed some identity to pestiviruses at the time of first analysis (2012), though a significant similarity to APPV was apparent once the genome of this pestivirus became available in GenBank (March 2015; [16]; Figure 1A). Via genome walking, the full length sequence of the viral genome was obtained (APPV NL1). The presence of the virus was confirmed by PCR in all 11 piglets obtained from the March and April 2012 serum samples of Farm 1 (Table 1). Additional blood samples (controls) were obtained from the index farm in July 2012; after clinical problems had diminished, one of the controls was PCR-positive (one out of 15).

Problems with outbreaks of congenital tremor continued on Farm 1 in 2013 and 2014. A smaller outbreak of the disease was diagnosed in January 2013. Four newborn precolostral piglets with clinical signs of congenital tremor type A-II were tested. The blood samples of these piglets again contained APPV NL1 (4/4 piglets). A second smaller outbreak of the disease was diagnosed in March 2013. Three newborn precolostral piglets were obtained for necropsy, all with congenital tremor type A-II. APPV NL1 strain was detected in 3/3 samples. One month later, in April 2013, four piglets were sampled in each of nine different healthy litters. None of these 36 control piglets carried APPV NL1. A third smaller outbreak occurred in January/February 2014. Seven piglets showing clinical signs of congenital tremor type A-II were analyzed, and APPV NL1 was detected in 7/7 serum samples.

An APPV NL1 qRT-PCR was performed on samples from the heart, small intestine, large intestine, brain, thoracic spinal cord, lumbar spinal cord, liver, inguinal lymph node, lung, gallbladder, urinary bladder, kidney, tonsil, spleen, serum and PBLs of seven necropsied precolostral newborn affected piglets. APPV NL1 could be detected in serum, PBLs and in all of the organs. The highest quantities were detected in tonsils, inguinal lymph nodes and serum. The same organs were sampled from control piglets from a farm with no history of congenital tremor type A-II (*n* = 7, Farm 9). All organs, PBLs and sera were APPV-negative.

During the 2013–2016 period, newborn piglets of several other farms with congenital tremor were sampled (Farms 2–8, 10, 11). A combination of VIDISCA analysis and PCR for the detection of APPV revealed that the virus circulates in farms in the Netherlands and in Spain (Farm 11) that reported outbreaks of congenital tremor. Table 1 shows the number of serum samples of piglets with congenital tremor tested on each farm, the number of APPV PCR-positive piglets and the strain name of the virus detected. In summary, all serum samples from piglets with disease (*n* = 99) were APPV PCR-positive. Furthermore, six of 66 (9%) control samples were PCR-positive (collected at Farms 1 and 3). The universal qRT-PCR described in the Materials and Methods section, which is based on amplification primers located in the 5′UTR, enabled the detection of all strains described in Table 1.

### 3.2. APPV Strains

At least one third of the genome could be determined for most APPV strains: from the start codon of the coding region until nucleotide 4428 in the ORF of the virus, except for APPV NL7 (1100 nt) and APPV NL5 (first 480 nt and nt 1977 till 4940 within the ORF). An identity matrix among the various strains and phylogenetic analysis revealed that there are clusters of strains representing different genotypes with intra-genotype diversity around 5%–7% and inter-genotype diversity >10% (Figure 1B, Table S2). We provisionally named the clusters genotypes A, B and C. To determine the variation among the strains along the genome, an identity plot was generated. This plot shows that, in the coding regions where we have genome information of most types, there are no strongly-conserved regions (Figure S2).

### 3.3. APPV Viremia in Blood and Fecal Shedding

We studied blood (serum) viremia and stool virus excretion of APPV early in life in 12 piglets (Farm 6, strain APPV NL7). All piglets had a detectable viremia in serum collected at their first week of life. Eleven of 12 (92%) congenital tremor piglets remained APPV NL7-positive in serum obtained three weeks after birth. The quantitative amount of virus in serum remained in the same order of magnitude over the three-week period (ranging between 10^3^ and 10^7^ RNA copies/mL). Ten of 15 (67%) shed APPV in stool in the first week of life and 10/12 (83%) at three weeks of age.

We monitored an additional 29 piglets of three affected litters in time to investigate prolonged virus shedding. Viral RNA copy numbers in serum remained in the order of 10^4^–10^6^ RNA copies per mL serum at six weeks and nine weeks of age, but the viral load reduced and the number of serum-positive pigs had declined at 4.5 months of age (seven of 20 pigs positive, on average 10^3^ RNA copies per mL serum; Table S3). During this period, clinical tremors had diminished in the majority of the affected pigs, but intensified in some of them during handling.

Shedding of the virus in stool was monitored for five additional pigs. Feces were collected at 6, 7, 7.5 and 8.5 months of age, and also, serum was analyzed. All five animals shed the virus in feces (PCR-positive; Table S4A). Interestingly, one male pig turned positive again in serum at 7.5 months of age (Table S4B). Preputial fluid of this same pig was collected from five months of age onwards, after the serum viremia had declined. Relatively high copy numbers of APPV could be detected in preputial fluid (10^4^–10^5^ RNA copies/mL) throughout the complete collection period (Table S5). Semen could not be collected from this animal, as it was not trained to ejaculate.

### 3.4. APPV in Serum and Feces of the Sow at Farrowing

We investigated if sows that gave birth to APPV-positive piglets were themselves positive for the virus in blood in the first days after farrowing. This was done to shed light on the moment of infection. None of the five sows with an affected litter contained APPV in serum at the time of delivery; only one sow had a rectal swab with detectable amounts of virus. This indicates that mother-to-child transmission of APPV occurs most probably during gestation, not during or shortly before farrowing, and trans-placental transmission of the virus is the most likely transmission route.

### 3.5. Inoculation of Pregnant Gilts and Congenital Tremor Type A-II in the Litters

Three gilts obtained from a high health farm were artificially inseminated. The sperm was checked for being APPV-negative by PCR. Pregnancy of all three gilts was confirmed at day 28 of gestation using ultrasound analysis. The gilts were infected via intramuscular inoculation with an inoculum of 0.22-µm filtrated APPV NL1-positive serum, containing 6 × 10^5^ copies of APPV RNA per mL (see Material and Methods section 2.8 and 2.9 for the preparation of the serum). At 10 days post-infection, three gilts were PCR-positive for the virus (measured in serum), although one of the three gilts showed relatively low viral loads (gilt 49: 5.5 × 10^2^ genome copies/mL versus 1.4 × 10^4^ copies/mL and 2.3 × 10^4^ copies/mL in gilts 50 and 51; Table 2). All gilts gave birth on day 114 or day 115 of gestation. At the time of farrowing, serum and stool samples of all three sows were PCR-negative for APPV. Piglets were scored for the presence of congenital tremor within hours after farrowing. Two of the three litters contained piglets showing clinical signs of congenital tremor; with mild, moderate, severe signs (11/13 and 13/15) or no clinical signs (two in each litter; Table 2). The moderate to severely-affected piglets presented tremor all over the body, including sometimes a shaking head, which was absent during inactivity. Mild tremor presented as a subtle tremor that did not result in overt shaking of the entire body, but fasciculation in the area of the hind limbs. Of note, abnormal numbers of piglets with splay leg syndrome were observed in litters 50 (*n* = 3) and 51 (*n* = 7). Several piglets in these litters presented as weak piglets with an abnormal posture (bended back (kyphosis) and ears on the neck). A PCR for APPV was performed, and 11 out of 13 and 15 out of 15 were PCR-positive for the virus (from gilts 50 and 51, respectively). Two piglets were PCR-negative, and these were the two litter 50 piglets without disease. Virus quantities in affected piglets were high and ranged in the order of 10^6^–10^7^ copies/mL, except for one piglet in litter 50 with mild tremor (PCR-positive, but not quantifiable, a concentration close to the detection level (<1000 RNA copies/mL serum; Table S6). We did not see a relationship between the virus concentration in blood and the severity of symptoms. Even two piglets without congenital tremor (in litter 51) contained high virus quantities in serum (Table S6).

Interestingly the litter of gilt 49 showed no congenital tremor, and all piglets of this litter, were APPV PCR-negative. This litter was born from the sow with low viremia at 10 days post-inoculation.

## 4. Discussion

APPV is a recently-discovered porcine pestivirus, first described by Hause et al. [16]. Soon thereafter, a link with congenital tremor type A-II was suggested by Arruda et al. [15] and Postel et al. [24]. The frequent detection and the large genetic variation of the virus in the Netherlands and Spain, in addition to the USA [15,16] and Germany [24,25], shows that APPV has probably spread worldwide. We measured a very strong association with congenital tremor. All 99 samples (100%) from piglets with disease sampled at 10 different farms were PCR-positive, whereas only six of 66 (9%) of the controls without clinical signs were positive. This strong association was also found by Arruda et al. [15]: 15/16 serum samples of congenital tremor piglets PCR-positive, four controls negative; and Postel et al. [24]: 6/6 congenital tremor serum sample positive, 2/2 controls PCR-negative.

Congenital tremor and APPV were further investigated using an animal model. We inoculated gilts with APPV at 32 days post-insemination. This timing was chosen to ensure the infection of the fetus prior to day 70 of gestation. Acquired immunity in pigs develops after day 70 of gestation [26], which was experimentally shown for Bungowannah pestivirus, where a humoral response was shown to occur only after in utero infection at day 70 or later [27]. A detectable high APPV-viremia was noted in two of our three gilts at day 10 post-inoculation. One gilt showed a 100-fold lower viremia at 10 days post-inoculation, compared to the other gilts, and thus, this gilt may not have experienced its first infection by the virus. Longitudinal blood sampling and testing could have shed light on the productivity of infection; however, we deliberately sampled the gilts only once after infection to avoid the possibility of miscarriage due to stress induced by repeated blood sampling. The piglets born from the gilt with low viremia were all congenital tremor-free and virus negative, whereas the two gilts with high levels of viremia delivered litters with piglets affected by tremors. The two affected litters showed some piglets with moderate to severe clinical signs of disease, a majority of piglets with mild clinical signs and even some with no clinical signs, similar to what we observe at farms with outbreaks. Our findings are in agreement with the findings by Arruda et al., who experimentally infected fetuses [15], whereas we infected pregnant gilts. Arruda et al. [15] also observed large variation in the severity of clinical signs within affected litters. The reason for this variation in clinical signs is unknown. In theory, the presence or absence of clinical signs may be related to the exact timing of infection in the fetus; the virus infection may spread from fetus to fetus, as was described for Bungowannah virus [27]. However, Arruda et al. [15] inoculated APPV at day 45 or day 62 post-gestation in the amniotic vesicles and noticed no obvious differences in disease severity of the offspring between the two inoculation dates.

A large number of strains was detected on the farms included in this study; in fact, almost each farm with clinical disease had a unique strain of the virus. As there are no rules for fine-tuning strains of pestivirus in separate genotypes, we arbitrarily chose the 10% distance as the border for type deviation, which was supported by phylogenetic clustering. The APPV strain described by Hause et al. [16] has a 13% distance with our strains and subsequently also represents a separate genotype, while the strain described by Arruda et al. [15] had a 9%–10% distance, and thus, it may be that this is a distant member of the A-type. It is important to state here that our distinction between types is solely based on genome analyses and not on serotype analysis or phenotype. We have to investigate if there are immunological differences between genotypes. Of note, all strains found during the years at Farm 1 are of the same type (APPV NL1), with some evolution during the years (Figure 1B). Our observation that persistently-infected asymptomatic carriers exist and that farm infections in time are of the same type explains why congenital tremor type A-II is often recurrent at the farm level, especially when new gilts are introduced in the population.

Hause et al. [16] presented an immune test that showed high frequency of infection in some farms, whereas one farm was without seropositive pigs. Unfortunately, we did not manage to set up a robust serological assay for APPV. For this reason, no information is available on the immunological status of the animals included in our study. Although PCR-negative for the virus, the gilts used in our study could have encountered APPV previously and developed immunity to the virus. In theory, gilt 49 that gave birth to healthy piglets could have had such pre-existing immunity. However, this should be tested further, together with vaccination experiments to see if protection can be established.

We observed splay leg and abnormal posture in the affected litters, in agreement with the findings of Arruda et al. [15], who also noted a high frequency (up to 40%) of splay leg in congenital tremor litters. These signs are general characteristics of weak piglets. We hypothesize that the virus may be circulating widely in the commercial pig population and causes not only congenital tremor type A-II, but also hitherto unexplained cases of weak piglets with abnormal posture and increased incidences of splay leg that currently go unexplained. Furthermore, a mild tremor may often go unnoticed or misdiagnosed as a sign of piglets shivering from cold.

We are very cautious about the interpretation of data related to the genotype of the infecting virus. We observed that Farm 3 had litters with APPV NL3 (genotype B) with congenital tremor, but also with APPV NL4 (genotype C) in two out of four litters without clinical signs, suggestive that this particular genotype C strain may be less pathogenic. However, this would be premature to conclude. Further analyses preferably need pure virus stocks after culturing and controlled infection, yet regrettably culture attempts have been unsuccessful so far. We inoculated several cell lines with serum or organ homogenates from APPV-positive piglets. The cell line panel included PK15 and SK6 (swine kidney cell lines) and primary swine kidney cells. No replication of the virus was detected in any of the cell lines tested. Similar approaches have been undertaken by Hause et al. [16] and Arruda et al. [15], also without success. Beer et al. [25] reported replication of APPV on porcine kidney cell line SPEV, cell line 0008, Collection of Cell Lines in Veterinary Medicine, FLI, but also mention that further analysis during passaging is necessary. Thus, it appears that infectivity of this virus in cell lines is limited, which may be related to the different structure of the E2 protein, noted by Hause et al. [16] and Beer et al. [25].

So far, the only study that located the virus at the site of the disease (the central nervous system) was performed by Postel et al. [24]. Fluorescent in situ hybridization showed strong staining of the inner granular cell layer of the cerebellum. We attempted immunostaining of the virus in the cerebellum, but did not manage to detect the virus due to high background staining. Postel et al. [24] suggested that distribution of virus within herds may occur via the orofecal route since a significant amount of APPV genome was present in salivary glands, duodenum, pancreas and colon. This is in accordance with our observation of frequent detection and prolonged shedding of the virus in stool.

In summary, piglets with clinical signs of congenital tremor type A-II undergo a long-lasting infection with APPV. A scenario with prolonged or even persistent carriers and shedders of the virus on affected farms is likely. Further studies to elucidate the exact routes of transmission at affected farms are necessary and future efforts should focus on virus control, e.g., detection and isolation of animals with disease and vaccination strategies. Such approaches have successfully been set up for other pestiviruses, such as classical swine fever virus, bovine viral diarrhea virus and border disease virus.

## Figures and Tables

**Table 1:** Presence and distribution of atypical porcine pestivirus (APPV) types.

Clinical Signs	Farm	No. of Samples	Date	# PCR-Positive Samples (Strain)	Type
CT *	1	6	March 2012	6 (APPV NL1) **	A
CT	1	5	April 2012	5 (APPV NL1)	A
CT	1	4	January 2013	4 (APPV NL1)	A
CT	1	3	March 2013	3 (APPV NL1)	A
CT	1	4	January 2014	4 (APPV NL1)	A
CT	1	3	February 2014	3 (APPV NL1)	A
CT	2	8	August 2013	8 (APPV NL2) **	C
CT	3	8	October 2013	8 (APPV NL3)	B
CT	4	5	May 2013	5 (APPV NL5)	B
CT	5	10	December 2013	10 (APPV NL6)	B
CT	6	15	January 2014	15 (APPV NL7)	C
CT	6	4	January 2014	4 (APPV NL7)	C
CT	7	4	March 2014	4 (APPV NL8) **	B
CT	8	4	February 2014	4 (APPV NL9)	C
CT	10	4	March 2016	4 (APPV NL11)	B
CT	11	12	February 2016	12 (APPV SP12)	A
CONTROLS ***	9	1	March 2013	0	
CONTROLS ***	9	6	December 2014	0	
CONTROLS	1	15	July 2012	1 (APPV NL1)	A
CONTROLS	3	8	October 2013	5 (APPV NL4)	C
CONTROLS	1	36	April 2013	0	

* CT = congenital tremor. ** Strains of which the sequence is used as a reference in type analysis; NL: Netherlands; SP: Spain. *** Collected on a farm with no history of congenital tremor.

**Table 2:** Congenital tremor type A-II in litters from APPV-infected gilts.

Gilt	APPV Viral Load in the Gilt *	Congenital Tremor Type A-II in the Offspring			APPV (pos/Tested) **
		Moderate/Severe Score 2	Mild Score 1	Absent Score 0	
**50**	1.4 × 10^4^	3	8	2	11/13
**51**	2.3 × 10^4^	1	12	2	15/15
**49**	5.8 × 10^2^	0	0	13	0/13

* RNA copies/mL in serum of the gilts 10 days post-experimental infection, average of duplicate PCR. ** Determined by PCR, pos: positive; see Table S6 for virus concentrations per animal.

## Supplementary Materials

The following are available online at www.mdpi.com/1999-4915/8/10/271/s1, **Figure S1**: Mean viral load in blood plasma of six weaning-aged piglets inoculated with tissue homogenate containing APPV; **Figure S2**: Similarity plot of APPV types B, C, APPV-000515 compared to type A; **Table S1**: Primers used for APPV PCRs; **Table S2**: Nucleotide distance matrix of the region between nt 379 (position start codon APPV type A) and nt 4803 of APPV; **Table S3**: APPV presence in serum at different ages; **Table S4**: APPV presence in feces at different ages; **Table S5**: APPV presence in preputial fluid; **Table S6**: APPV RNA load in offspring of experimentally-infected gilts.
