# Supplementary material for: Atypical Porcine Pestivirus: A Possible Cause of Congenital Tremor Type A-II in Newborn Piglets
Source: Viruses. 2016 Oct 4;8(10):271. doi: 10.3390/v8100271 (PMC5086607; doi:10.3390/v8100271)
Supplement: Supplementary file 1 [file viruses-08-00271-s001.docx]

Supplementary Materials: Atypical Porcine Pestivirus-A Possible Cause of Congenital Tremor Type A-II in Newborn Piglets

Ad de Groof, Martin Deijs, Lars Guelen, Lotte van Grinsven, Laura van Os-Galdos, Wannes Vogels, Carmen Derks, Toine Cruijsen, Victor Geurts, Mieke Vrijenhoek, Janneke Suijskens, Peter van Doorn, Leo van Leengoed, Carla Schrier and Lia van der Hoek

**Table S1.** Primers used for atypical porcine pestivirus (APPV) PCRs.

| **Name** | **DNA Sequence** | **Position in Reference Genome** |
| --- | --- | --- |
| F2 | 5′-CGGATACAGAAATACTAC-3′ | 10206–10223 |
| R2 | 5′-CCGAATGCAGCTARCAGAGG-3′ | 10521–10540 |
| F1 | 5′-GCCATGATGGAGGAAGTG-3′ | 10263–10280 |
| R1 | 5′-GGGCAGRTTTGTGGATTCAG-3′ | 10399–10418 |
| F3 | 5′-GAGTACGGGGCAGACGTCAC-3′ | 163–182 |
| R3 | 5′-CATCCGCCGGCACTCTATCAAGCAG-3′ | 320–344 |
| F4 | 5′-ATGCATAATGCTTTGATTGG-3′ | 1–20 |
| R4 | 5′-GTGACGTCTGCCCCGTACTC-3′ | 163–182 |
| F3B | 5′-CGTGCCCAAAGAGAAATCGG-3′ | 247–266 |
| R3B | 5′-CCGGCACTCTATCAAGCAGT-3′ | 317–336 |

**Table S2.** Nucleotide distance matrix of the region between nt 379 (position start codon APPV) and nt 4803.

| **Strain/Cluster** | **A** | **A** | **B** | **B** | **B** | **B** | **C** | **C** | **C** |  |  |
| --- | --- | --- | --- | --- | --- | --- | --- | --- | --- | --- | --- |
| Name | CT-APPV-1 | CT-APPV-12 | CT-APPV-3 | CT-APPV-6 | CT-APPV-8 | CT-APPV-11 | CT-APPV-2 | CT-APPV-4 | CT-APPV-9 | APPV000515 | APPV-ISUVDL |
| APPV NL1 | 0.0000 | 0.0349 | 0.1037 | 0.1024 | 0.1019 | 0.1041 | 0.1011 | 0.1015 | 0.1018 | 0.1373 | 0.0917 |
| APPV SP12 | 0.0349 | 0.0000 | 0.1077 | 0.1048 | 0.1038 | 0.1060 | 0.1025 | 0.1059 | 0.1078 | 0.1452 | 0.1002 |
| APPV NL3 | 0.1037 | 0.1077 | 0.0000 | 0.0616 | 0.0594 | 0.0634 | 0.0990 | 0.1002 | 0.1047 | 0.1417 | 0.1073 |
| APPV NL6 | 0.1024 | 0.1048 | 0.0616 | 0.0000 | 0.0041 | 0.0073 | 0.1038 | 0.1045 | 0.1073 | 0.1390 | 0.1050 |
| APPV NL8 | 0.1019 | 0.1038 | 0.0594 | 0.0041 | 0.0000 | 0.0050 | 0.1020 | 0.1019 | 0.1047 | 0.1368 | 0.1029 |
| APPV NL11 | 0.1041 | 0.1060 | 0.0634 | 0.0073 | 0.0050 | 0.0000 | 0.1039 | 0.1051 | 0.1063 | 0.1397 | 0.1051 |
| APPV NL2 | 0.1011 | 0.1025 | 0.0990 | 0.1038 | 0.1020 | 0.1039 | 0.0000 | 0.0688 | 0.0659 | 0.1337 | 0.0993 |
| APPV NL4 | 0.1015 | 0.1059 | 0.1002 | 0.1045 | 0.1019 | 0.1051 | 0.0688 | 0.0000 | 0.0706 | 0.1328 | 0.1035 |
| APPV NL9 | 0.1018 | 0.1078 | 0.1047 | 0.1073 | 0.1047 | 0.1063 | 0.0659 | 0.0706 | 0.0000 | 0.1366 | 0.1054 |
| APPV 000515 | 0.1373 | 0.1452 | 0.1417 | 0.1390 | 0.1368 | 0.1397 | 0.1337 | 0.1328 | 0.1366 | 0.0000 | 0.1398 |
| APPV ISUDVD | 0.0917 | 0.1002 | 0.1073 | 0.1050 | 0.1029 | 0.1051 | 0.0993 | 0.1035 | 0.1054 | 0.1398 | 0.0000 |

**Table S3.** APPV presence in serum at different ages.

A: APPV RNA in pigs at 6 weeks of age

| **Gilt** | **Litter Information** | | **Serum APPV Copy Number/mL** | | |
| --- | --- | --- | --- | --- | --- |
|  | **# Piglets** | **# APPV-Positive** | **Copies (Average)** | **(Highest)** | **(Lowest)** |
| 1 | 10 | 10 | 2.1 × 10^5^ | 5.1 × 10^5^ | 5.7 × 10^4^ |
| 2 | 10 | 10 | 1.1 × 10^5^ | 7.5 × 10^5^ | 5.4 × 10^3^ |
| 3 | 9 | 9 | 7.4 × 10^5^ | 2.2 × 10^5^ | 6.8 × 10^4^ |

B: APPV RNA in pigs at 9 weeks of age

| **Gilt** | **Litter Information** | | **Serum APPV Copy Number/mL** | | |
| --- | --- | --- | --- | --- | --- |
|  | **# Piglets** | **# APPV-Positive** | **Copies (Average)** | **(Highest)** | **(Lowest)** |
| 1 | 10 | 10 | 7.9 × 10^5^ | 1.6 × 10^6^ | 2.6 × 10^4^ |
| 2 | 10 | 10 | 7.0 × 10^5^ | 2.1 × 10^6^ | 4.8 × 10^4^ |
| 3 | 7 | 7 | 5.5 × 10^5^ | 1.4 × 10^6^ | 1.4 × 10^4^ |

C: APPV RNA in pigs at 4.5 months of age

| **Gilt** | **Litter Information** | | **Serum APPV Copy Number/mL** | | |
| --- | --- | --- | --- | --- | --- |
|  | **# Piglets** | **# APPV-Positive** | **Copies (Average)** | **(Highest)** | **(Lowest)** |
| 1 | 10 | 3 | 7.4 × 10^3^ | 1.0 × 10^4^ | 1.4 × 10^3^ |
| 2 | 4 | 1 | 1.8 × 10^3^ | 1.8 × 10^3^ | 1.8 × 10^3^ |
| 3 | 6 | 3 | 5.2 × 10^3^ | 8.6 × 10^3^ | 1.8 × 10^3^ |

**Table S4.** APPV presence in feces at different ages.

A

|  |  | **Shedding APPV in Feces (Age in Months)** | | | |
| --- | --- | --- | --- | --- | --- |
| **Pig** | **Boar/Gilt** | **6** | **7** | **7.5** | **8.5** |
| 87 | Gilt | POS | POS | POS | POS |
| 92 | Gilt | POS | POS | ND | POS |
| 93 | Gilt | POS | POS | POS | POS |
| 100 | Gilt | POS | POS | ND | ND |
| 152 | Boar | POS | POS | POS | POS |

ND: not detectable; POS: positive (no quantitative analyses due to unknown dilution factor).

B

|  | | **Serum*** | | | | | | |
| --- | --- | --- | --- | --- | --- | --- | --- | --- |
|  |  | **APPV Copy Number/mL** | | | | | | |
| **Pig** | **Boar/Gilt** | **6 Weeks** | **9 Weeks** | **4.5 Months** | **6 Months** | **7 Months** | **7.5 Months** | **8.5 Months** |
| 87 | Gilt | 6.8 × 10^4^ | 1.8 × 10^6^ | 1.2 × 10^3^ | ND | ND | ND | ND |
| 92 | Gilt | 1.6 × 10^4^ | 2.2 × 10^5^ | ND | ND | ND | ND | ND |
| 93 | Gilt | 1.7 × 10^4^ | 4.8 × 10^4^ | ND | ND | ND | ND | ND |
| 100 | Gilt | 7.1 × 10^4^ | 5.7 × 10^5^ | 9.0 × 10^3^ | ND | ND | ND | ND |
| 152 | Boar | 4.4 × 10^4^ | NA | 4.7 × 10^4^ | ND | ND | 1.5 × 10^3^ | ND |

*Serum of pigs from Table S4A; ND: not detectable; NA: not analyzed.

**Table S5.** APPV presence in preputial fluid.

|  |  | **Preputial Fluid APPV Copy Number/mL (Age in Months)** | | | | | |
| --- | --- | --- | --- | --- | --- | --- | --- |
| **Pig** | **Boar/Gilt** | **6** | **6.5** | **7** | **7.5** | **8** | **8.5** |
| 152 | Boar | 1.7 × 10^5^ | 1.5 × 10^6^ | 1.8 × 10^5^ | 8.1 × 10^4^ | 1.1 × 10^3^ | 4.1 × 10^4^ |

**Table S6.** APPV RNA load in offspring of experimentally infected gilts.

| **Gilt** | **Piglet Ear-tag** | **Tremor** | **APPV RNA Copies/mL** | **Remarks** | **Splay Leg** |
| --- | --- | --- | --- | --- | --- |
| 49 | 181 | 0 | neg |  |  |
|  | 182 | 0 | neg |  |  |
|  | 183 | 0 | neg |  |  |
|  | 184 | 0 | neg |  |  |
|  | 185 | 0 | neg |  |  |
|  | 186 | 0 | neg |  | Y |
|  | 187 | 0 | neg |  |  |
|  | 188 | 0 | neg |  |  |
|  | 189 | 0 | neg |  |  |
|  | 190 | 0 | neg |  |  |
|  | 191 | 0 | neg |  |  |
|  | 192 | 0 | neg |  |  |
|  | 193 | 0 | neg |  |  |
|  | 194 |  |  | dead, not scored |  |
| **Gilt** | **Piglet Ear-tag** | **Tremor** | **APPV RNA Copies/mL** | **Remarks** | **Splay Leg** |
| 50 | 201 | 2 | 2.12 × 10^7^ |  |  |
|  | 202 | 1 | 2.88 × 10^7^ |  |  |
|  | 203 | 1 | 1.98 × 10^6^ |  |  |
|  | 204 | 1 | 6.92 × 10^6^ |  |  |
|  | 205 | 2 | 2.61 × 10^7^ |  |  |
|  | 206 | 1 | 1.33 × 10^7^ |  | Y |
|  | 207 | 0 | neg |  |  |
|  | 208 | 0 | neg |  |  |
|  | 209 | 1 | Pos: <1 × 10^3^ |  |  |
|  | 210 | 2 | 1.77 × 10^6^ |  |  |
|  | 211 | 1 | 9.05 × 10^5^ |  | Y |
|  | 212 | 1 | 1.56 × 10^7^ |  | Y |
|  | 213 | 1 | 1.04 × 10^6^ |  |  |
|  | 214 |  |  | dead, not scored |  |
|  | 215 |  |  | dead, not scored |  |
|  | 216 |  |  | dead, not scored |  |
| **Gilt** | **Piglet Ear-tag** | **Tremor** | **APPV RNA Copies/mL** | **Remarks** | **Splay Leg** |
| 51 | 221 | 1 | 1.04 × 10^7^ |  | Y |
|  | 222 | 1 | 1.37 × 10^7^ |  | Y |
|  | 223 | 1 | 7.45 × 10^6^ |  | Y |
|  | 224 | 0 | 7.88 × 10^6^ |  |  |
|  | 225 | 1 | 6.34 × 10^6^ |  |  |
|  | 226 | 1 | 2.42 × 10^6^ |  |  |
|  | 227 | 2 | 5.32 × 10^6^ |  | Y |
|  | 228 | 1 | 4.70 × 10^6^ |  |  |
|  | 229 | 1 | 9.03 × 10^6^ |  |  |
|  | 230 | 1 | 2.18 × 10^7^ |  |  |
|  | 231 | 0 | 2.23 × 10^7^ |  | Y |
|  | 232 | 1 | 9.51 × 10^6^ |  |  |
|  | 233 | 1 | 3.31 × 10^6^ |  |  |
|  | 234 | 1 | 2.11 × 10^6^ |  | Y |
|  | 235 | 1 | 1.83 × 10^7^ |  | Y |

Score:

0: no recognizable tremor

1: mild tremor

2: moderate/severe tremor

Y: splay leg syndrome

**Figure S1.** Mean viral load in blood plasma of six weaning-aged piglets inoculated with tissue homogenate containing APPV. X-axis: days post-inoculation; Y-axis: mean viral load in plasma in RNA copies/mL. The arrow indicates Day 11, the day the serum used to inoculate the gilts was collected. The lower detection limit of the real time PCR was 1000 copies/mL. On day 0 and day 3, the mean viral load was below the detection limit. Average viral load Day 10: 2.8 × 10^5^ (*n* = 6;
SEM 2.7 × 10^4^); average viral load Day 14: 2.7 × 10^6^ (*n* = 3; SEM 2.2 × 10^5^).

**Figure S2.** Similarity plot of APPV types B, C and APPV compared to APPV type A**.** The similarity plot of the region between nucleotide 379 (position start codon APPV type A) and nucleotide 4803 was generated via Simplot, with APPV type A (Farm1_15MAR2012) as the reference and APPV-8 (Farm7_MAR2014) and APPV-2 (Farm2_AUG2013) as representatives of types B and C respectively. GenBank Entry KR011347 was used for APPV (Hause et al., 2015, J Gen Virol; 96,
2994–2998) [16].
